# Multidimensional-Constrained Suspect Screening of Hydrophobic Contaminants Using Gas Chromatography-Atmospheric Pressure Chemical Ionization-Ion Mobility-Mass Spectrometry

**DOI:** 10.1021/acs.analchem.4c06234

**Published:** 2025-03-06

**Authors:** Xiaodi Shi, Anna Sobek, Jonathan P. Benskin

**Affiliations:** Department of Environmental Science, Stockholm University, Stockholm 10691, Sweden

## Abstract

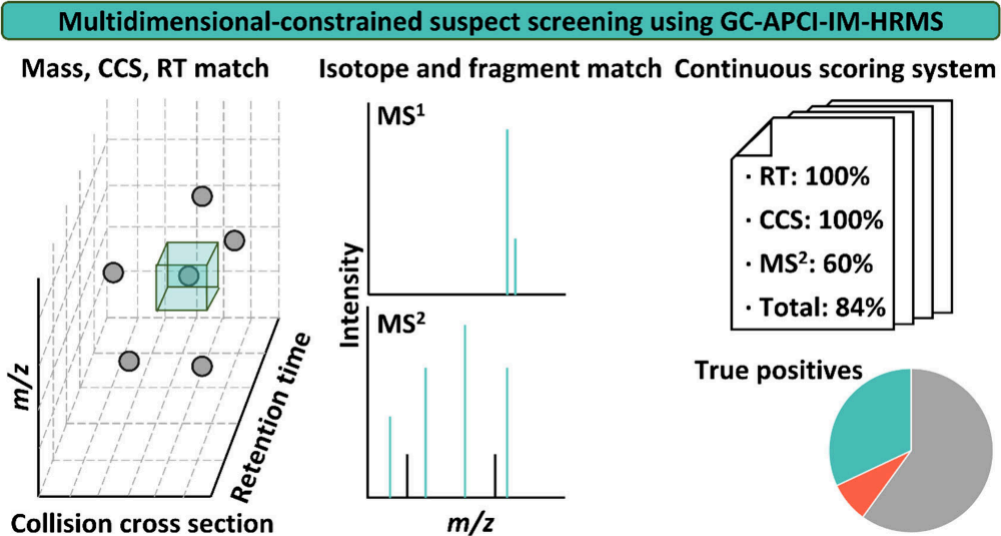

Suspect screening strives to rapidly monitor a large number of substances in a sample using mass spectral libraries. For hydrophobic organic contaminants (HOCs), these libraries are traditionally based on electron ionization mass spectra. However, with the growing use of state-of-the-art mass spectrometers, which often use alternative ionization approaches and separation techniques, new suspect screening workflows and libraries are urgently needed. This study established a new suspect screening library for 1,590 HOCs, including exact mass and a combination of measured and model-predicted values for retention time (RT) and collision cross section (CCS). The accuracy of *in silico* predictions was assessed using standards for 102 HOCs. Thereafter, using gas chromatography-atmospheric pressure chemical ionization-ion mobility-mass spectrometry, a suspect screening workflow constrained by the full scan mass spectrum of (quasi-)molecular ions (including isotope patterns), RT, CCS, and fragmentation mass spectra, together with a continuous scoring system, was established to reduce false positives and improve identification confidence. Application of the method to fortified and standard reference sediment samples demonstrated true positive rates of 79% and 64%, respectively, with all false positives attributed to suspect isomers. This study offers a new workflow for improved suspect screening of HOCs using multidimensional information and highlights the need to enrich mass spectral databases and extend the applicable chemical space of current *in silico* tools to hydrophobic substances.

## Introduction

The number of chemicals used in society is large and constantly increasing. For instance, a survey of 22 chemical inventories from 19 countries revealed over 350,000 registered substances,^1^ while between 700 and 1,700 new chemicals are registered in the United States and European Union every year.^2^ To improve the capacity for chemical monitoring and risk assessment, suspect screening has emerged as a tool for rapidly detecting a large number of substances by matching data acquired by high-resolution mass spectrometry (HRMS) to that contained in databases. High-confidence matches guide the acquisition of standards for the final confirmation or exclusion of an identification.^3^

The conventional approach to suspect screening of hydrophobic organic contaminants (HOCs) involves matching experimental and library retention indices (RIs) and mass spectra derived from gas chromatography–mass spectrometry (GC-MS) analysis using electron ionization (EI) at 70 eV.^4^ However, the efficacy of this approach tends to be limited by the availability of library mass spectra.^3^ Moreover, in the absence of a library match, *de novo* structure elucidation is hampered by extensive fragmentation and the frequent absence of molecular ions using EI. To address these limitations, new approaches utilizing softer ionization and alternative separation techniques are needed, together with highly specific suspect screening lists and workflows.

Atmospheric pressure chemical ionization (APCI), a soft ionization technique for GC, can preserve molecular ions for HOCs through charge/proton transfer.^5^ When coupled to ion mobility (IM)-HRMS, coeluting ions can be further separated based on their interactions with neutral buffering gases under an electric field to enhance the resolving-power of product ion scans. Collision cross section (CCS) values derived from IM are a novel instrument-independent property of ions for identification.^6^ The combination of CCS, full scan mass spectrum (MS^1^ – including both [quasi-]molecular ion exact mass and isotope patterns), retention time (RT), and fragmentation mass spectrum (MS^2^) represents multidimensional information that can be acquired or predicted for each compound, thereby improving confidence in structure assignments. Nevertheless, suspect screening of HOCs using multidimensional information has not been fully explored, with the exception of a few studies focused on establishing CCS databases.^6,7^

This study aimed to establish and validate a multidimensional-constrained workflow for suspect screening of HOCs using GC-APCI-IM-HRMS. Suspect lists were compiled consisting of exact mass and a combination of measured and model-predicted RT and CCS values. A continuous scoring system was established to narrow down the number of candidates. Finally, the method was validated using sediment samples, due to their complexity as a matrix and function as a sink for HOCs.

## Materials and Methods

### Instrumental Analysis

The present work used a Waters quadrupole-cyclic ion mobility-time-of-flight mass spectrometer (Waters Corp., Wilmslow, U.K.) coupled to an Agilent 8890 GC (Agilent Technologies, Santa Clara, CA, U.S.A) via an APCI source. The instrument was operated in positive ionization mode under both dry and wet conditions using previously optimized parameters.^8^ The detector was operated in high-definition MS^E^ mode with the collision energy fixed at 6 eV in low energy mode and ramped between 15 and 50 eV in high energy mode. The detailed instrumental method is described in **Section A** of the Supporting Information (SI). Progenesis QI (version 3.0, Waters Corp.) was used for lockmass correction, precursor and product ion pairing, peak picking, and alignment. MS^2^ data were exported from Progenesis QI and converted to SIRIUS format using a custom R script (available at https://github.com/Xiaodi-Shi/4D-suspect). Software parameters are provided in **Section B** of the SI.

### Suspect Lists

We compiled two suspect lists containing identity (i.e., CAS, InChIKey, and formula), exact mass, RT, and CCS for a total of 1,590 substances. The first list (1,060 substances) was derived from matching contaminants from all GC-based suspect lists in the NORMAN Suspect List Exchange^9^ with experimentally derived CCS values from a unified database for environmental contaminants.^6^ The second list (530 substances) was derived from both the Arctic Monitoring and Assessment Programme’s list of chemicals of emerging Arctic concern, as well as the European Chemicals Agency’s list of substances of very high concern, after removing substances with ionizable functional groups and duplicate substances.^10,11^

Due to ionization through charge/proton transfer using APCI,^5^ the exact mass of the most abundant isotope for both M^+.^ and [M + H]^+^ ions was calculated. Based on a prior study showing that the CCS difference for M^+.^ and [M + H]^+^ ions tended to be less than 2%,^7^ we assumed that possible CCS differences among M^+.^, [M + H]^+^, and [M-H]^−^ ions fell within measurement uncertainty. Predictions for CCS values of [M + H]^+^ ions were made for 456 contaminants lacking literature CCS values using the AllCCS2 algorithm.^12^ Since alkanes evade ionization using APCI, the Fiehn RI was adopted with fatty acid methyl esters as references.^13^ Experimental Fiehn RIs and Kovats RIs were collected from the MS-DIAL metabolomics MSP spectral kit and the NIST RI library, respectively, if available.^14^ Kovats RIs for the remaining 978 compounds were predicted using a deep convolutional neural network method.^15^ Kovats RIs were converted to Fiehn RIs, and thereafter RT. Converting between RT and RI is described in **Section C** and **Tables S1–3** in the SI. The two suspect lists can be found in Tables S4 and S5.

The quality of suspect list data was estimated by comparing measured and literature/model-predicted values. MS^1^, RT, CCS, and MS^2^ were acquired for 102 HOC standards (1.44 < log *K*_ow_ < 16.8; −9.31 < log *K*_aw_ < 11.3), including polycyclic aromatic hydrocarbons (PAHs), organophosphate esters (OPEs), polychlorinated biphenyls (PCBs), polybrominated diphenyl ethers (PBDEs), and other organohalogens. We further predicted RTs for all 102 of these contaminants and CCS values for a subset of 85 capable of generating M^+.^ or [M ± H]^+^ ions as base peaks using aforementioned models. Measured data for these contaminants is listed in Table S6, while comparisons between measured and reference values in suspect lists are listed in Table S7.

### Multidimensional Suspect Screening

Inspired by a previously developed system for metabolomics,^16^ we established a multidimensional (i.e., MS^1^, RT, CCS, and MS^2^) workflow for HOC suspect screening. In the MS^1^ dimension, both exact mass and isotope pattern matches for (quasi-)molecular ions were mandatory, and continuous scores were calculated for RT, CCS, and MS^2^. Unlike confidence level systems (e.g., the Schymanski confidence scale),^17^ the continuous scoring system enables ranking of candidates based on their scores, thereby reducing false positives. For exact mass measurements, the error threshold was set to <5 ppm for substances with mass-to-charge >200 Da and <2 mDa for substances <200 Da.^3^ Observation of at least one isotope ion was required, and the geometric mean of isotopic relative intensity deviation was required to be <5%.^18^

RT and CCS scores were derived using Equations (Eqs) 1–3. Differences (Δ) between measured (i.e., *RT*_*measured*_ or *CCS*_*measured*_) and reference (i.e., *RT*_*reference*_ or *CCS*_*reference*_) values were calculated using eqs 1 and 2. *ΔRTs* or *ΔCCS* values within the high-confidence threshold (*CT*_*high*_) scored 100%, while peaks with *ΔRTs* or *ΔCCS* values outside the low-confidence threshold (*CT*_*low*_) were excluded. Continuous scores were calculated using eq 3 for *ΔRTs* or *ΔCCS* values between *CT*_*high*_ and *CT*_*low*_. The thresholds were determined based on comparing measured and literature/model-predicted values in suspect lists (see discussion in the next section for the threshold values). The custom R script for matching peaks against suspect lists by exact mass, RT, and CCS is available at https://github.com/Xiaodi-Shi/4D-suspect.
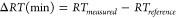
1
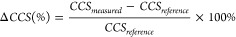
2
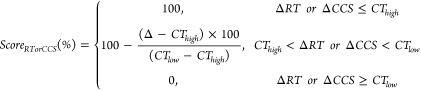
3MS^2^ similarity scores were acquired from SIRIUS+CSI:FingerID (version 5.8.6).^19^ Since candidates were known after matches of MS^1^, RT, and CCS, we specified the formula, adduct, parent ion, and InChIKey in SIRIUS (see **Section B** of the SI for SIRIUS parameters). The performance of SIRIUS was evaluated by scoring measured MS^2^ data from available HOC standards. A total of 63 out of 102 measured contaminants produced M^+.^/[M + H]^+^ ions as the base peak, with MS^2^ spectra containing at least two monoisotopic fragment ions. Fragment ions of these 63 contaminants are provided as SI in NIST MSP library format.

The final multidimensional score was calculated using the weighed (*W*) sum of individual scores for RT, CCS, and MS^2^ (eq 4).
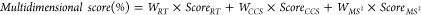
4The true positive rate was independent of weightings in this study, based on testing with real sediment samples (Figure S1). Considering the dependence of RT on chromatographic conditions, less weight was assigned to the RT score (*W*_*RT*_ = 0.2) than CCS and MS^2^ scores (both *W*_*CCS*_ and *W*_*MS2*_ = 0.4).

When the MS^2^ score could not be estimated (e.g., <two detected monoisotopic fragment ions in MS^2^), accurate measurement of RT and CCS becomes essential. Thus, the cutoff for the multidimensional score was set at 60% for potential candidates. The highest-scoring candidate for each suspect compound was retained.

### Workflow Validation

The true positive identification rate was assessed by characterizing two types of sediment (both measured in triplicate) for 37 contaminants (Table S7) which occurred on our suspect lists and for which standards were available. The first sediment was a NIST standard reference material (SRM; 1941b-Organics in Marine Sediment), while the second was a fortified surface (0–2 cm) sediment collected from a reference site (58.26° N, 16.91° E) in the Baltic Sea. The NIST material was used as-received, while the fortified sediment was prepared by addition of approximately 1 ng of each contaminant. Samples were extracted by accelerated solvent extraction (ASE 350; Dionex, U.S.A.) using acetone/*n*-hexane (1:1 v/v), following previously described procedures (see **Section D** of the SI for details).^8^ Instrumental analysis and data processing were carried out as described above.

## Results and Discussion

### Performance of Model Predictions for Suspect Lists

**Figure 1:**
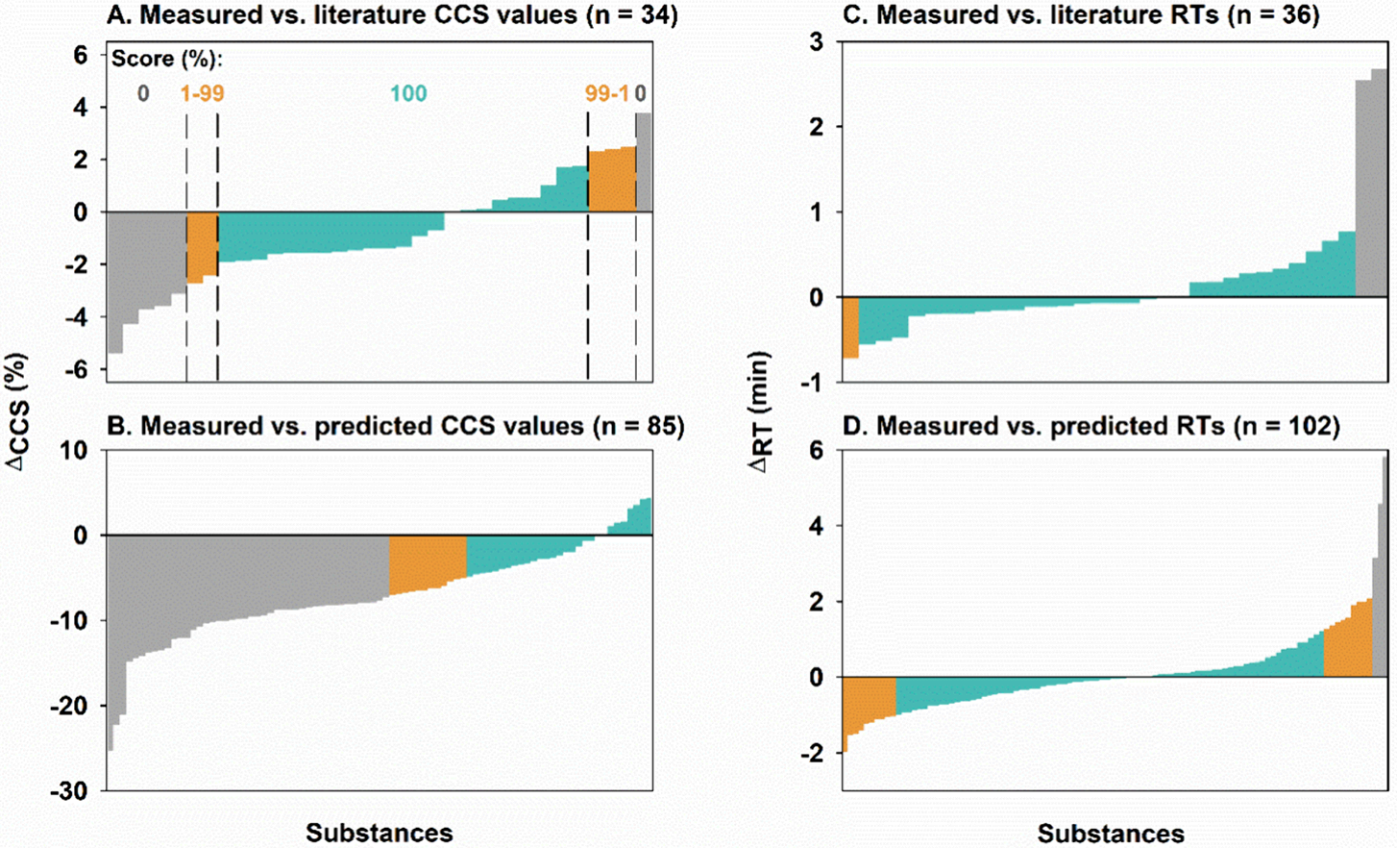
Distribution of relative errors (%) between measured collision cross section (CCS) values and either literature (A) or predicted (B) values, as well as the distribution of differences between measured retention times (RTs) and either literature (C) or predicted (D) values. Using Panel A as an example, differences within high-confidence thresholds (green bars) score 100%, while differences outside low-confidence thresholds (gray bars) score 0%. A continuous score was calculated for differences between high- and low-confidence thresholds (orange bars). n is the number of substances.

Among 102 measured contaminants, 34 already had literature CCS values in our suspect lists, offering an opportunity to evaluate our measurement accuracy. Previous interlaboratory and interplatform comparisons reported that CCS relative errors tend to be within ±2%.^6,7,20,21^ In the present work, *ΔCCS* between measured and experimentally derived values in our suspect lists fell within ±2% for 67.6% of substances, and within ±3% for 82.3% (Figure 1A). Similar uncertainties were also observed when comparing our measurements to previous measurements acquired using a similar instrumental technique (Figure S2).^7^ Based on these results, *CT*_*high*_ and *CT*_*low*_ for literature CCS values were set at ±2% and ±3%, respectively. Experimentally determined CCS values for 53 substances are reported here for the first time (Table S6).

The average *ΔCCS* between our measured and predicted values is −7.03% (standard deviation [SD]: ± 5.42%), indicating an overprediction of CCS values by the ALLCCS2 model. This considerable discrepancy resulted from the fact that most compounds used for model training are nonhalogenated endogenous metabolites which tend to have larger CCS values than halogenated HOCs.^12,22^ All relative differences in measured CCS values among available isomers fell within 5%. *CT*_*high*_ and *CT*_*low*_ for predicted CCS values were set at ±5% and ±7% covering 34.1% and 48.2% of 85 measured contaminants, respectively, to avoid misidentification at the isomeric level (Figure 1B).

Uncertainties associated with RT predictions (mean ± SD of 0.086 ± 1.13 min, n = 102) are comparable to those of RTs converted from literature RI values (mean ± SD of 0.15 ± 0.69 min, n = 36; Figure 1C,D), due to the similarity between our measured contaminants and compounds for model training.^15^ For RTs from both sources, mean ± SD and mean ±2 × SD were selected as *CT*_*high*_ and *CT*_*low*_, respectively.

The mean ± SD of SIRIUS-derived MS^2^ scores for 63 known HOCs was 72.4 ± 18.0% (see Table S7 for detailed values). Although this model is mainly trained on electrospray ionization data (i.e., polar substances),^19^ the high scores indicate that it is also applicable to HOCs and that the mechanism of collision induced dissociation is independent of ionization method.

### Workflow Validation

**Figure 2:**
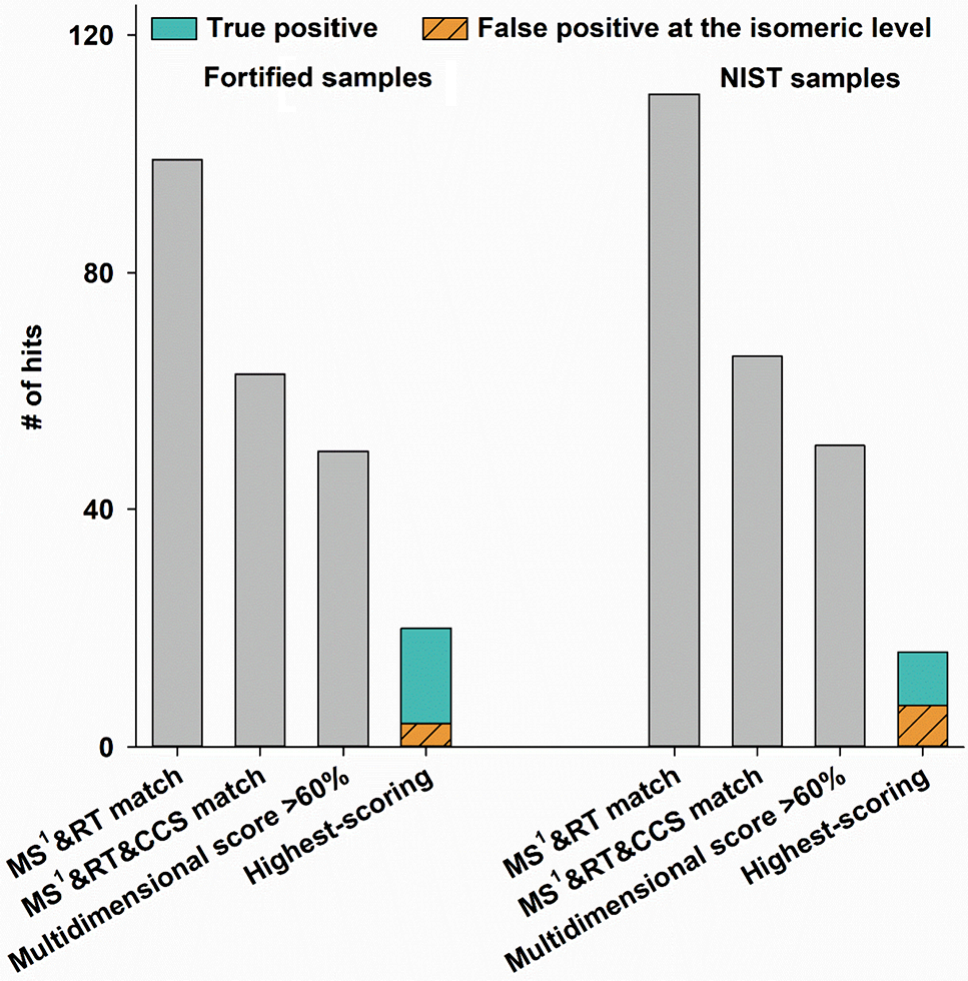
Number of hits in fortified and NIST samples at different steps in the suspect screening workflow (from left to right): hits with full scan mass spectrum (MS^1^ – both (quasi)molecular ion exact mass and isotope patterns) and retention time (RT) match; MS^1^, RT, and collision cross section (CCS) match; multidimensional score over 60%; and the highest score.

There were 63 and 66 hits in the fortified sediment and SRM 1941b, respectively, with matches in three dimensions (i.e., MS^1^, RT, and CCS; Figure 2). These numbers are 36.3% and 40.0% lower than those obtained with only MS^1^ and RT matched, respectively, indicating that the analytical space for potential candidates can be effectively constrained using CCS as an additional dimension.

After checking the MS^2^, 50 candidates in the fortified sediment and 51 candidates in SRM 1941b were retained (multidimensional scores >60%; Figure 2). According to the Schymanski confidence scale,^17^ these candidates would be classified as level 2/3. Finally, the highest-scoring candidates for each suspect contaminant were retained (19 in the fortified sediment and 14 in SRM 1941b), including PAHs, PCBs, PBDEs, OPEs, hexachlorobenzene, and 1,2-bis(2,4,6-tribromophenoxy)ethane (see Table S8 for detailed scores), resulting in true positive rates of 78.9% and 64.3%, respectively (Figure 2). Notably, all false positives were isomers of the correct compounds. These results suggest that the combination of MS^1^ and MS^2^ data can effectively determine the elemental composition, particularly for halogenated compounds, while CCS further constrains the general structure.

### Perspectives

Based on analytical data acquired by GC-APCI-IM-HRMS, a suspect screening workflow for HOCs constrained by MS^1^, RT, CCS, and MS^2^, together with a continuous scoring system, was established for the first time. The true positive rate of ∼70% was achieved with misidentification only at the isomeric level. Highly reproducible CCS values for the (quasi-)molecular ion represents a confirmatory dimension which improves identification confidence. However, due to the frequent absence of molecular ions when using a more conventional GC-EI-MS approach, most existing experimental CCS values are only available for compounds suitable for electrospray ionization (i.e., polar substances). Our suspect lists contain literature CCS values for 1,134 HOCs, representing only 28% of contaminants in the unified CCS database,^6^ and over 20 times fewer than the number of compounds in the METLIN-CCS database.^23^ Consequently, current CCS prediction models displayed considerable error (mean of −7.23%) when predicting CCS for HOCs (which are mainly halogenated) in this study. Preservation of molecular ions using APCI presents opportunities to enrich mass spectral databases for HOCs and to extend the applicable chemical space of current CCS-prediction models to HOCs.

## Supporting Information Available

MS^2^ library (TXT)
Suspect lists (Tables S4–S5); measured analytical data for hydrophobic organic contaminant standards (Table S6); evaluation of data quality of suspect lists and prediction model performances (Table S7); highest-scoring candidates in sediment (Table S8) (XLSX)
Instrumental method (Section A); software parameters (Section B); conversions among RTs and RIs (Section C and Tables S1–S3); true positive rates using different weights (Figure S1); sample preparation (Section D); relative error distribution between measured and literature CCS values (Figure S2) (PDF)
