# Supplementary material for: Multidimensional-Constrained Suspect Screening of Hydrophobic Contaminants Using Gas Chromatography-Atmospheric Pressure Chemical Ionization-Ion Mobility-Mass Spectrometry
Source: Anal Chem. 2025 Mar 6;97(10):5434–8. doi: 10.1021/acs.analchem.4c06234 (PMC11923942; doi:10.1021/acs.analchem.4c06234)
Supplement: Supplementary file 3 — ac4c06234_si_003.pdf [file ac4c06234_si_003.pdf]

Supporting Information

Multidimensional-constrained Suspect Screening of Hydrophobic Contaminants Using Gas Chromatography-Atmospheric Pressure Chemical Ionization-Ion Mobility-Mass Spectrometry

Xiaodi Shi[1]*, Anna Sobek[1], Jonathan P. Benskin[1]

1. Department of Environmental Science, Stockholm University, Stockholm 10691, Sweden

* Address correspondence to Xiaodi Shi: Department of Environmental Science, Stockholm University, Stockholm 10691, Sweden. E-mail: xiaodi.shi@aces.su.se. Telephone: 46-73-891 2864. ORCID: 0009-0008-4062-4009

Summary: 11 pages, texts, 8 tables, 2 figures, and references.

Contents:

Section A. Instrumental method.

Section B. Software parameters.

Section C. Conversions among retention times and retention indices.

Table S1. Regressions among retention times and retention indices.

Table S2. Retention times and retention indices of fatty acid methyl esters.

Table S3. Measured retention times of alkanes.

Table S4. Suspect list of gas-chromatography-amenable compounds with experimentally-derived collision cross section values. (Supporting Excel file)

Table S5. Suspect list of chemicals of concern. (Supporting Excel file)

Table S6. Measured analytical data for hydrophobic organic contaminant standards. (Supporting Excel file)

Table S7. Evaluation of data quality of suspect lists and prediction model performances. (Supporting Excel file)

Figure S1. Variation of true positive rate (%) using different weights.

Section D. Sample preparation.

Figure S2. Distribution of relative errors between measured collision cross section values with values reported by Izquierdo-Sandoval et al.

Table S8. Highest-scoring candidates in sediment samples. (Supporting Excel file)

References

Section A. Instrumental method. A Waters quadrupole-cyclic ion mobility-time-of-flight mass spectrometer (Waters Corp., Wilmslow, U.K.) coupled to an Agilent 8890 GC (Agilent Technologies, Santa Clara, CA, U.S.A) via APCI was employed for analysis. 1 μL of sample was injected in pulse splitless mode with a programmed inlet temperature for vaporization (i.e., initially 100 °C for 0.15 min, increased at 600 °C min^{-1} to 280 °C, and held for 1 min). Analytes were separated by a 30-m DB-5MS Ultra Inert column (i.d., 0.25 mm; film thickness, 0.25 μm; Agilent Technologies) with helium carrier gas at a constant flow of 1.5 mL min^{-1}. The GC oven temperature program was as follows: held at 70 °C for 1 min; increased at 10 °C min^{-1} to 310 °C, and then held for 15 min.

The APCI source was operated in positive mode. The transfer line and ion source were maintained at 290 °C and 150 °C, respectively. The corona discharge and cone voltage were set at 2 μA and 30 V, respectively. Nitrogen was used as the makeup, auxiliary, and cone gas at flow rates of 200 mL min^{-1}, 350 L h^{-1}, and 250 L h^{-1}, respectively, under dry conditions. An uncapped bottle of water was placed in the ionization enclosure to achieve under wet conditions. Auxiliary and cone gas flowes were reduced to 150 and 200 L h^{-1}, respectively, under wet conditions.

The mass detector was operated in high-definition MS^E mode with a mass range of 100-1200 amu. The collision energy was fixed at 6 eV in low energy mode, and ramped between 15-50 eV in high energy mode. The scan time was 0.3 s for each mode. The IM cell was operated in one pass mode with 3 pushes per bin at a traveling wave height of 22 V. Both drift and collision gas were nitrogen. Column bleeding ($C_9H_{27}O_5Si_5^+$: m/z 355.0705) was measured every 2 min for internal mass calibration. CCS was calibrated using a mixture of 22 compounds supplied by Waters Corp. according to their standard procedure.

Section B. Software parameters. Progenesis QI (version 3.0, Waters Corp., Wilmslow, U.K.) was used for peak-picking and alignment, with the sensitivity threshold of 50. To simplify data, all ions in MS^1 were considered as $M^{+\cdot}$. In MS^2, fragment ions with intensities lower than 1% of parent ions were excluded.

SIRIUS+CSI:FingerID (version 5.8.6) was used to estimate MS^2 similarity.[1] For APCI, analytes can generate $M^{+\cdot}$ or $[M+H]^+$ ions through charge or proton transfer, respectively. Although both adduct forms can be chosen, the parent ion in SIRIUS has to be the protonated ion. Therefore, a mass of hydrogen (i.e., 1.0078 Da) was added to each parent ion, before input. Since candidates were known after MS^1, RT, and CCS match, we specified the formula, adduct, parent ion, and InChIKey. Default values were used for other parameters.

Section C. Conversions among retention times and retention indices. We employed methods described by Kind et al.[2] To convert between measured RT using GC-APCI and Fiehn RI, fifth-grade polynomial regressions (i.e., Equation 1 and 2 in Table S1) were established based on fatty acid methyl esters (FAMEs). Measured RT and Fiehn RI for FAMEs are listed in the Table S2.

To convert from reference Kovats to Fiehn RIs, both n-alkanes and FAMEs were measured using GC-EI-MS in selective ion monitoring mode. The temperature programs were identical for both instruments. The quantitative ions for n-alkanes and FAMEs were mass-to-charge ratios of 57 and 87, respectively. Measured RT for FAMEs and n-alkanes using EI are listed in Table S2 and S3, respectively. Reference Kovats RIs were converted to observed Kovats RI using a linear regression based on FAMEs (i.e., Equation 3 in Table S1). Observed Kovats RIs were further converted to Fiehn RIs using a fifth-grade polynomial regression based on FAMEs (i.e., Equation 4 in Table S1).

Table S1. Regressions among retention times and retention indices.

Regression	R^2	Equation
Fiehn RI = $-1.761e^{-7} \times (RT)^4 + 7.989e^{-4} \times (RT)^3 - 1.336 \times (RT)^2 + 1659 \times (RT) - 282600$	1	1
RT (sec.) = $1.219e^{-21} \times (\text{Fiehn RI})^4 - 3.673e^{-15} \times (\text{Fiehn RI})^3 + 4.076e^{-9} \times (\text{Fiehn RI})^2 - 5.092e^{-4} \times (\text{Fiehn RI}) + 380.7$	1	2
Obs. Kovats RI = 1.0230356 x ref. Kovats RI	1	3
Fiehn RI = $5.498e^{-9} \times (\text{Obs. Kovats RI})^4 - 3.427e^{-5} \times (\text{Obs. Kovats RI})^3 - 0.01264 \times (\text{Obs. Kovats RI})^2 + 682.9 \times (\text{Obs. Kovats RI}) - 430600$	1	4

Abbreviation: RI: Retention index; RT: Retention time; Obs.: observed; Ref.: Reference

Table S2. Retention times and retention indices of fatty acid methyl esters.

Substance	Composition	Retention time (s)		Kovats retention index		Fiehn retention index
		Atmospheric pressure chemical ionization	Electron ionization	Reference	Observed	
Methyl laurate	$C_{13}H_{26}O_2$	744	704	1481	1521	487220
Methyl myristate	$C_{15}H_{30}O_2$	882	843	1680	1723	582620
Methyl palmitate	$C_{17}H_{34}O_2$	1008	970	1878	1924	668720
Methyl stearate	$C_{19}H_{38}O_2$	1124	1091	2077	2135	747420
Methyl arachidate	$C_{21}H_{42}O_2$	1229	1191	2276	2325	819620
Methyl behenate	$C_{23}H_{46}O_2$	1327	1289	2475	2526	886620
Methyl tetracosanoate	$C_{25}H_{50}O_2$	1418	1381	2674	2732	948820
Methyl hexacosanoate	$C_{27}H_{54}O_2$	1503	1470	2872	2943	1006900
Methyl octacosanoate	$C_{29}H_{58}O_2$	1588	1546	3071	3134	1061700

Table S3. Measured retention times of n-alkanes.

Substance	Composition	Retention time (s)	Kovats retention index
Pentadecane	$C_{15}H_{32}$	688	1500
Hexadecane	$C_{16}H_{34}$	760	1600
Heptadecane	$C_{17}H_{36}$	829	1700
Octadecane	$C_{18}H_{38}$	894	1800
Nonadecane	$C_{19}H_{40}$	956	1900
Icosane	$C_{20}H_{42}$	1015	2000
Heneicosane	$C_{21}H_{44}$	1072	2100
Docosane	$C_{22}H_{46}$	1126	2200
Tricosane	$C_{23}H_{48}$	1178	2300
Tetracosane	$C_{24}H_{50}$	1229	2400
Pentacosane	$C_{25}H_{52}$	1277	2500
Hexacosane	$C_{26}H_{54}$	1323	2600
Heptacosane	$C_{27}H_{56}$	1367	2700
Octacosane	$C_{28}H_{58}$	1411	2800
Nonacosane	$C_{29}H_{60}$	1453	2900
Triacontane	$C_{30}H_{62}$	1493	3000
Hentriacontane	$C_{31}H_{64}$	1532	3100
Dotriacontane	$C_{32}H_{66}$	1573	3200
Tritriacontane	$C_{33}H_{68}$	1619	3300
Tetratriacontane	$C_{34}H_{70}$	1673	3400

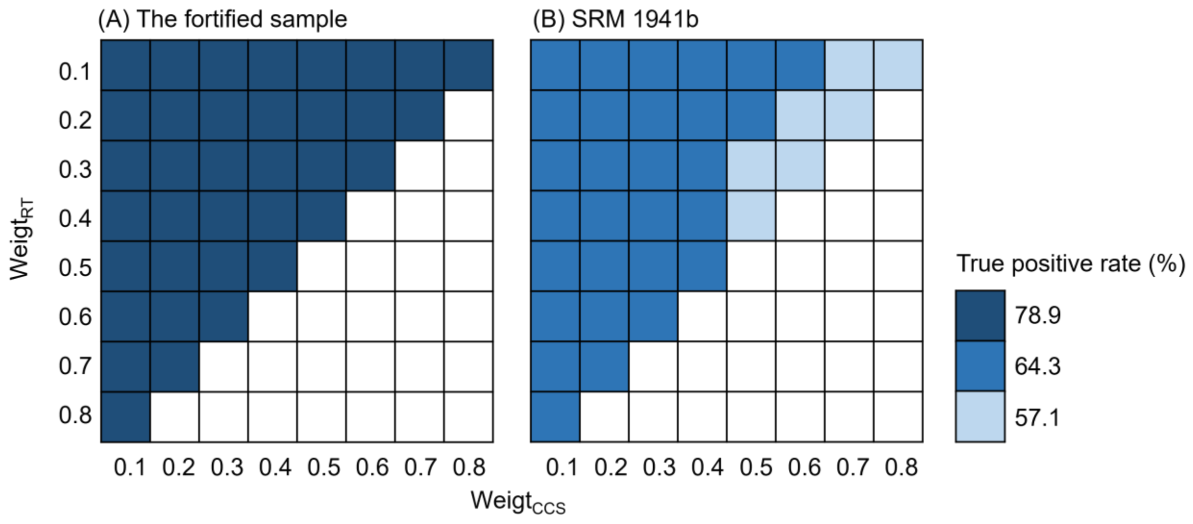

Figure S1. True positive rates (%) using different weights. RT: retention time; CCS: collision cross section. $Weight_{SIRIUS} = 1 - Weight_{RT} - Weight_{CCS}$.

Section D. Sample preparation. Extraction was performed using an accelerated solvent extraction system (ASE 350; Dionex, U.S.A). 34-mL extraction cells were pre-cleaned using 20 mL of ACE/HEX (1:1 v/v) at 100 °C for 10 min. Empty cells were dried in a clean fume hood at room temperature overnight. Cells were loaded sequentially with a pre-baked glass-fiber filter (GFF; Dionex, Thermo Scientific), diatomaceous earth (DE; Dionex, Thermo Scientific), approximately 4 g of freeze-dried sediment, DE, and a GFF. Surface sediment from the Baltic Sea was fortified with both native and labeled standards before extraction, while NIST sediment was only fortified with labeled standards. Samples were extracted three times, each time with 20 mL of ACE/HEX (1:1 v/v) at 100 °C for 10 min. The system was washed three times with 5 mL of solvent each time between two samples. Extracts were concentrated to about 1 mL using a rotary evaporator at 30 °C, and transferred to a clean test tube. 1 mL of active copper in HEX was added to remove sulfur. After vortexing and centrifugation, supernatant was collected into a clean brown bottle, blew down with nitrogen to 200 µL, and transferred to a sample vial. All glassware and GFFs were baked at 450 °C for 4 h. All metal parts and tools were cleaned using an ultrasonic bath in ACE for 20 min. Potential photolysis was prevented by wrapping the containers with aluminum foil or using amber glassware.

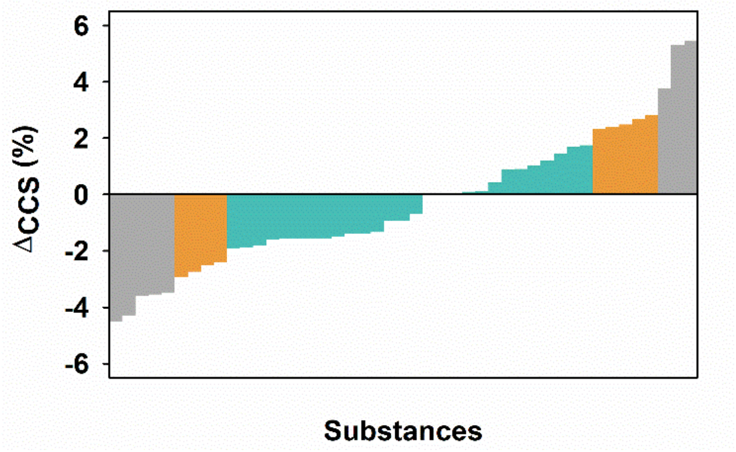

Figure S2. Distribution of relative errors (*ΔCCS*; %) between our measured collision cross section values with those reported by Izquierdo-Sandoval et al.[3] Green bars: *ΔCCS* <±2%; orange bars: ±2%< *ΔCCS* <±3%; gray bars: *ΔCCS* >±3%.

References

(1) Dührkop, K.; Fleischauer, M.; Ludwig, M.; Aksenov, A.; Melnik, A.; Meusel, M.; Dorrestein, P.; Rousu, J.; Böcker, S. SIRIUS 4: A rapid tool for turning tandem mass spectra into metabolite structure information. *Nat. Methods.* **2019**, *16*, 299–302.

(2) Kind, T.; Wohlgemuth, G.; Lee, D.; Lu, Y.; Palazoglu, M.; Shahbaz, S.; Fiehn, O. FiehnLib: Mass spectral and retention index libraries for metabolomics based on quadrupole and time-of-flight gas chromatography/mass Spectrometry. *Anal. Chem.* **2009**, *81*, 10038–10048.

(3) Izquierdo-Sandoval, D.; Fabregat-Safont, D.; Lacalle-Bergeron, L.; Sancho, J.; Hernández, F.; Portoles, T. Benefits of ion mobility separation in GC-APCI-HRMS screening: From the construction of a CCS library to the application to real-world samples. *Anal. Chem.* **2022**, *94*, 9040–9047.
